# Three-dimensional midwater camouflage from a novel two-component photonic structure in hatchetfish skin

**DOI:** 10.1098/rsif.2016.1034

**Published:** 2017-05-03

**Authors:** Eric I. Rosenthal, Amanda L. Holt, Alison M. Sweeney

**Affiliations:** Department of Physics and Astronomy, University of Pennsylvania, Philadelphia, PA 19104, USA

**Keywords:** camouflage, biophotonics, optical modelling, midwater ecology, bioluminescence

## Abstract

The largest habitat by volume on Earth is the oceanic midwater, which is also one of the least understood in terms of animal ecology. The organisms here exhibit a spectacular array of optical adaptations for living in a visual void that have only barely begun to be described. We describe a complex pattern of broadband scattering from the skin of *Argyropelecus* sp., a hatchetfish found in the mesopelagic zone of the world's oceans. Hatchetfish skin superficially resembles the unpolished side of aluminium foil, but on closer inspection contains a complex composite array of subwavelength-scale dielectric structures. The superficial layer of this array contains dielectric stacks that are rectangular in cross-section, while the deeper layer contains dielectric bundles that are elliptical in cross-section; the cells in both layers have their longest dimension running parallel to the dorsal–ventral axis of the fish. Using the finite-difference time-domain approach and photographic radiometry, we explored the structural origins of this scattering behaviour and its environmental consequences. When the fish's flank is illuminated from an arbitrary incident angle, a portion of the scattered light exits in an arc parallel to the fish's anterior–posterior axis. Simultaneously, some incident light is also scattered downwards through the complex birefringent skin structure and exits from the ventral photophores. We show that this complex scattering pattern will provide camouflage simultaneously against the horizontal radially symmetric solar radiance in this habitat, and the predatory bioluminescent searchlights that are common here. The structure also directs light incident on the flank of the fish into the downwelling, silhouette-hiding counter-illumination of the ventral photophores.

## 1. Introduction

**Figure 1.**
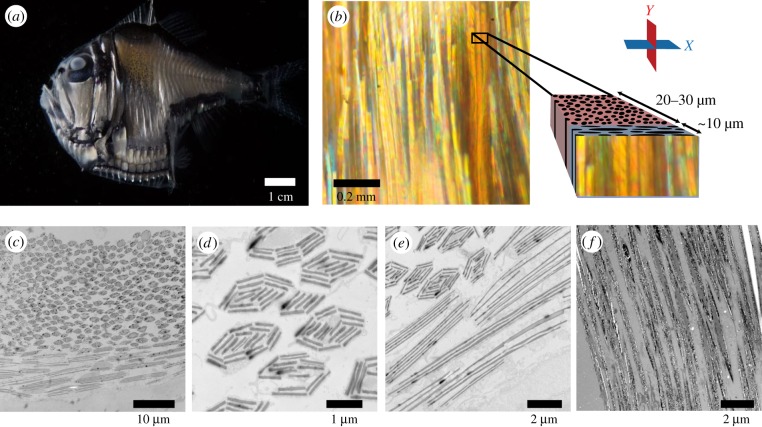
Images of hatchetfish and photonic skin structures. (*a*) Photograph of the lateral aspect of a hatchetfish showing the general anatomy including ventral photophores, and appearance of the fish in diffuse lighting. (*b*) Darkfield reflected-light micrograph of hatchetfish skin and diagram of cell types within the skin. Individual skin cells appear as reflective wire-like structures with long axes running vertically. Exploded view shows the relative orientation of the two distinct layers and cell types within the skin, and also with respect to the *X*- and *Y*-planes used to describe and model this structure. (*c*) Overview transmission electron micrograph (TEM) showing both layers of skin cells sectioned in the *X*-plane. (*d*) Detail TEM of the elliptical-bundle cells of the deeper layer, sectioned in the *X*-plane. (*e*) Detail TEM of the rectangular-bundle cells of the superficial skin layer. (*f*) Detail TEM of both layers of the skin sectioned in the *Y*-plane, showing that both cell types shown in cross-section in the *X*-plane are tens of microns long when sectioned along their longest axis.

Fish have evolved complex reflective structures in their skin for the purpose of camouflage [1,2]. Strategies for camouflage vary depending on a given species' depth range and the correlated environmental optical conditions [3–6]. Hatchetfish live in the mesopelagic zone of the ocean, between approximately 300 and 1000 m deep where irradiance is 5–10 orders of magnitude dimmer than at the surface [7,8]. As solar light propagates down from the ocean surface, it is scattered and absorbed such that, at mesopelagic depths, ambient light is 1–2 orders of magnitude brighter travelling in the downward direction than horizontally, while horizontal travelling light is radially symmetric [9]. Hatchetfish have an extremely laterally compressed body with broad reflective sides and a reduced tail, resembling the blade of a hatchet (figure 1*a*). The laterally compressed body, together with a line of ventral bioluminescent organs, simultaneously minimizes the downward projected area of the fish and matches the radiance of downwelling light, providing counter-illuminating camouflage from upward-looking predators hunting for prey silhouettes [10]. While the intensity of side-welling radiance in this mesopelagic region is low compared with downwelling radiance, previous work has shown that mirror-like specular reflectance may be a camouflage strategy against the radially symmetric side-welling radiance for animals in this habitat [1,4]. Prior to this study, hatchetfish were informally presumed to fall into this category of organisms using specular reflectance to hide against this radially symmetric horizontal radiance. However, as Zylinski and Johnsen have recently shown, animals are likely to require mechanisms for hiding both within low-intensity ambient radiance (via transparency in the case of cranchiid squid) as well as from the comparatively bright, directed beams of predatory bioluminescent searchlights (for instance, by deploying absorbing chromatophores over the transparent surface of the same squid) [11,12]. To our knowledge, it remains uninvestigated how reflective materials could provide camouflage against searchlights.

**Figure 2.**
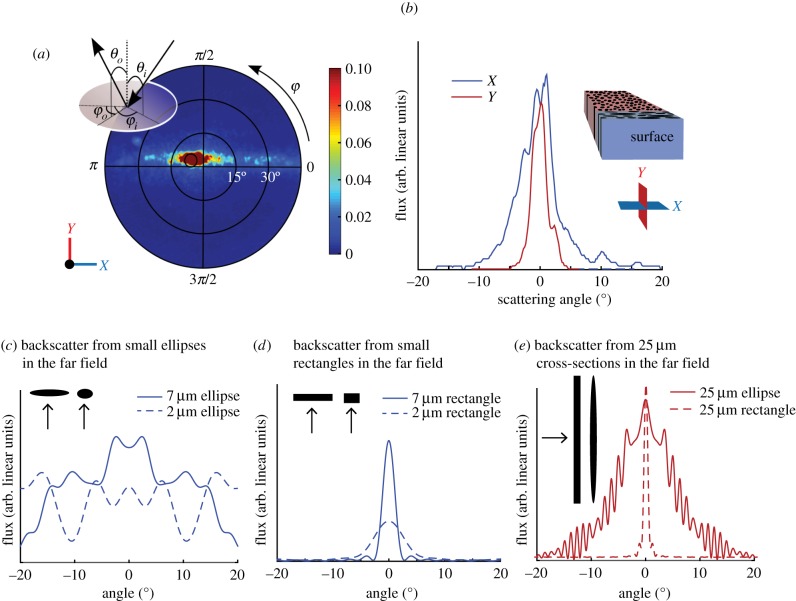
Reflectance distribution functions for hatchetfish skin and related structures. (*a*) Bidirectional reflectance distribution function (BRDF) of hatchetfish skin and definition of incident and scattering angles, as measured by Haag *et al*. [14]. Units along the intensity axis are arbitrary, and remain arbitrary in all panels in this figure. Small black circle shows the direction of the incident beam. (*b*) Reflectance distributions of hatchetfish skin along planes parallel and perpendicular to the ‘scattering arc’ where most reflected energy is directed by the fish skin, approximately described by *θ_o_* = −20° to 20° and *φ_o_* = 0° or 180° (blue line), and *θ_o_* = −20° to 20° and *φ_o_* = 90° or 270° (red line). (*c*) Backscattering from ellipses similar in size and shape to the *X* cross-sectional planes of cells found in hatchetfish skin. Dashed lines show backscatter from 2 µm long ellipses, analogous to backscatter from the cells in the deeper layer. Solid lines show backscatter from 7 µm long ellipses, analogous to backscatter from superficial-layer cells. (*d*) Backscattering from rectangles similar in size and shape to the *X* cross-sectional planes of cells found in hatchetfish skin. Dashed lines show backscatter from 2 µm long ellipses, analogous to backscatter from the cells in the deeper layer. Solid lines show backscatter from 7 µm long ellipses, analogous to backscatter from superficial-layer cells in the top layer. Integrated backscatter is roughly the same intensity as in panel (*c*). (*e*) Backscattering from rectangles and ellipses similar in size and shape to the cells sectioned along the *Y*-plane in both superficial and deep layers. Dashed lines show backscatter from 25 µm long rectangles, and solid lines show backscatter from 25 µm long ellipses.

Haag and colleagues [14] have shown that hatchetfish skin exhibits a significant departure from specular reflection. This work characterized the bidirectional reflectance distribution function (BRDF) of the skin. BRDF of a surface, defined at a specific wavelength, is the ratio of outgoing radiance *dL*_*o*_ to incoming radiance *dE*_*i*_ as described with respect to the coordinate system in figure 2*a*:1.1
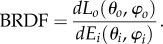

This characterization used a 532 nm source and incident angles of *θ* = 8º, 20º and 30º. This measurement showed that, for a beam directed at an arbitrary angle onto the flank of the fish, light is scattered into an arc parallel to the anterior–posterior axis of the fish, but at an angle consistent with specular reflectance defined with respect to the dorsal–ventral axis. This anisotropic scattering pattern must originate in the structured, high-index materials within the fish skin scale cells. Guanine crystals are the major refractive component of these scale cells. These crystals are flat platelets reported to have anisotropic refractive index with the shortest axis of the crystal corresponding to the lowest index axis [2,13,15–16]. In the absence of bulk metallic materials, structured and/or specular scattering in biological systems is typically achieved by the use of dielectric Bragg stacks (layers of material with average spacing less than the wavelength of light that create constructive interference and reflection of some wavelengths) [13,15,16]. Broadband reflection can be achieved by introducing variation in the spacing between high-index layers, or in high-index thicknesses, and has evolved many times in organisms such as beetles, squid and other fish [2,17–25].

Any consideration of midwater camouflage must also consider the predators there; many midwater predators use bioluminescent searchbeams, emitted from bioluminescent organs typically located near the eye, for locating prey. Bioluminescent flashes from animals in this region of the ocean emit between 10^7^ and 10^13^ photons at about 450 nm and are visible by other animals at distances of approximately 10–100 m away [26,27]. Bioluminescent flashes from searchlight beams must first scatter off the prey before bouncing back and reaching the predator's eye, requiring that two times the distance between the predator and prey be traversed by the beam before it is perceived by the predator. The black or red pigments found in many mesopelagic animals are presumably used to camouflage against predatory searchbeams by absorbing all incident light [28]. However, the metallic-appearing hatchetfish does not have an immediately apparent mechanism for hiding against searchlights found at similar depths and light levels.

Here, we used transmission electron microscopy (TEM) and optical modelling to determine the physical origins of this scattering behaviour, and to examine how the complex scattering contributes to open-water camouflage in the presence of searchlight predators. TEM revealed a surprisingly complex photonic structure consisting of two distinct layers: an outer layer of disordered Bragg stacks similar to those previously described in fish skin, and a thicker inner layer of approximately 2 µm major-axis elliptical bundles running parallel to the dorsoventral axis of the fish (figure 1*b–f*; [20,24]). The specific angular dependence of light scattered from the flank of the fish is the result of the orientation of these two distinct layers. The inner layer of the structure also apparently allows light to be guided downwards inside the animal's body and out of the ventral photophores. We show how the scattering from this complex structure contributes to camouflage in all viewing orientations in both the ambient solar radiance of the midwater environment and against the predatory searchlights that are a prevalent feature of ecological interactions here. To our knowledge, this is the first study to use a numerical approach to quantify how scattering from fish scales contributes to camouflage, and the first to investigate how reflecting rather than absorbing and transmitting materials contribute to camouflage from searchbeams.

## 2. Methods

### 2.1. Specimen collection

We collected specimens of *Argyropelecus* sp. at depths between 300 and 1000 m using a Mother Tucker trawl net with a thermally protecting cod end [29] on two separate cruises, one on the *R/V Endeavor* off the coast of Rhode Island, USA, in August 2014, and a second on the *R/V Hugh R. Sharp* off the Delaware coast, USA, in July 2016. Animals of size sufficient for optical characterization (length greater than 2 cm) were removed promptly from the trawl bucket, placed individually in cold, fresh seawater in plastic containers, stored at 4°C, and used in experiments within 24 h of collection. All measurements other than electron microscopy were performed in shipboard laboratories on freshly caught specimens.

Hatchetfish of species *A. aculeatus, A. sladeni, A. gigas* and *A. olfersi* were collected opportunistically from these trawls. They were used interchangeably in the various measurements described here, and, to the best of our ability to discern, the phenomena we describe are general to most if not all Sternoptychinae as a whole. However, further work will be required to determine whether there are subtler variations between species in the scale structures described here and the corresponding strategies for camouflage.

### 2.2. Transmission electron microscopy

Pieces of hatchetfish skin, approximately 3 × 3 mm in dimension, were dissected from the central portion of the flank of the animal. The dissected pieces were embedded in low-viscosity Spurr's resin according to the manufacturer's instructions (Electron Microscopy Sciences, Hatfield, PA, USA). Ultrathin (approx. 80 nm) sections were cut using a Leica microtome (Leica Biosystems, Nussloch, Germany). Sections were cut from adjoining orthogonal faces of the same resin block, resulting in perpendicular sectioning planes of the same region of skin. One sectioning plane was parallel to the anterior–posterior axis of the fish (figure 1, ‘plane *X*’) and the other sectioning plane was parallel to the dorsal–ventral axis of the fish (figure 1, ‘plane *Y*’). By imaging sections from these two intersecting planes, we were able to build two- and quasi-three-dimensional models of refractive structures in the skin. Sections were imaged using a JEOL transmission electron microscope (JEOL Ltd, Tokyo, Japan).

### 2.3. Computational electrodynamics model

We created two-dimensional and three-dimensional models of scattering from refractive structures within the fish skin by converting TEM images from the *X*-plane and *Y*-plane, as defined in figure 1*b*, into binary images where black pixels represent high-index guanine and white pixels represent low-index cytoplasm and extracellular space. To calculate scattering from both individual cells and groups of cells we used the finite-difference time-domain (FDTD) method. This method has previously been used to study photonic structures in marine organisms [20,25,30–32]. We used the MIT Electromagnetic Equation Propagator (MEEP) software for all FDTD calculations [33]. Scattering from individual cells and simple geometric shapes were performed in two dimensions while scattering from a section of fish tissue was performed in three dimensions using the NSF Extreme Science and Engineering Discovery Environment (XSEDE) supercomputer resource. All scattering simulations used an incident wavelength of 532 nm unless otherwise specified.

Previous studies of fish skin have modelled guanine crystals as isotropic, with a refractive index of *n* = (1.83,1.83,1.83), or birefringent, with a refractive index of *n* = (1.83,1.83,1.46) [20,34–36]. In this study, for simplicity, we assumed the isotropic model for guanine, combined with a low-index material of *n* = 1.33, approximately that of water and biological fluids.

**Figure 3.**
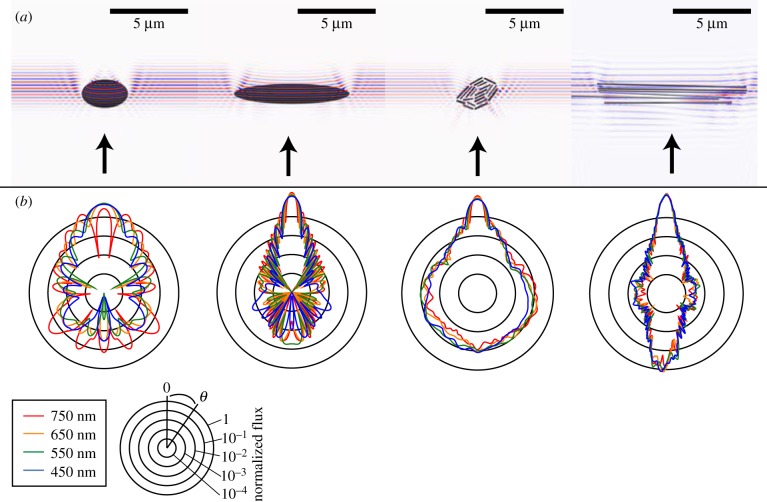
FDTD calculations of scattering as a function of wavelength from cross-sections of ellipses and complex cells from the superficial and deep layers of hatchetfish skin. (*a*) Snapshots of electric fields as calculated by FDTD interacting with cross-sections of shapes, analogous to the cells in hatchetfish skin and with cross-sections of the subwavelength structures present in these cells. Arrows show the angle of incidence of the plane-wave source. From left to right: elliptical cross-section analogous to deep-layer cells in the *X*-plane; elliptical cross-section analogous to superficial-layer cells in the *X*-plane; cross-section of the subcellular structure of a deep-layer cell; cross-section of the subcellular structure of a superficial-layer cell. (*b*) Wavelength-dependent phase functions of the structures in (*a*). Curves show radial coordinates of scattering intensity as calculated by the FDTD method at 750 nm (red), 650 nm (orange), 550 nm (green), and 450 nm (blue).

To understand how the complex scattering properties of hatchetfish skin are related to the hierarchical structure of the skin, we calculated phase functions for isolated structural features of the skin. We first modelled individual cells found within the composite skin structure as simple ellipses or rectangles with a homogeneous refractive index (figures 2*c*,*d* and 3). For the inner layer, the mean major (minor) axis of the elliptical cross-section of an individual cell was 2.94 (1.81) µm. For the outer layer, the same mean major (minor) axis of an individual cell was 7.34 (1.28) µm. The long axis of these homogeneous cells was set to 25 µm. These calculations therefore approximated backscattering from single cells along the *Y*-plane (figure 2*e*).

We then used MEEP to calculate two-dimensional phase functions for the literal cross-sectional structures observed in TEM when sectioned parallel to the *X*-plane (figure 3). For single-cell models with grid dimensions greater than 50 mm (figure 2*e*), we used the near2far function in MEEP to find scattering in the far field from these structures covering the same angular range probed by the BRDF instrument [14]. Otherwise, we had sufficient computational resources to reach the far-field regime for scattering in all directions. In all single-cell models (figures 2*c*–*e* and 3*e*–*h*), we averaged the scattering from three to five individual cells to reduce the amplitude of the scattering oscillations and determine the overall shape of the angular scattering.

**Figure 4.**
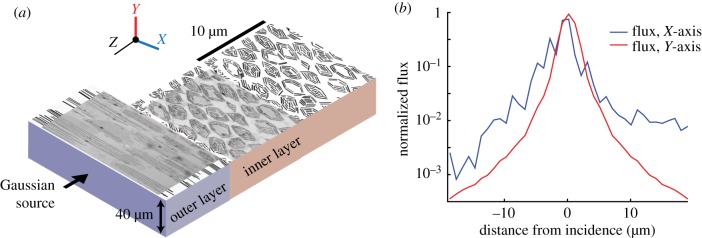
Three-dimensional FDTD analysis of the full complexity hatchetfish skin structure. (*a*) Schematic showing TEM images, binary image representations of hatchetfish skin structure, and spatial dimensions of the coordinates used in our FDTD model. In the model, a Gaussian source (width 1 µm) was incident on the plane indicated. (*b*) Flux calculated along detection planes placed parallel to the *X*-axis as defined in (*a*) (blue line) and along the *Y*-axis as defined in (*a*) (red line). Even in the near field, flux is more widely distributed along the *X*-axis than along the *Y*-axis, and is in general consistent with multiple scattering from the two-dimensional structures examined above.

Then, we used FDTD to calculate scattering from a three-dimensional model of a literal representation of field of these cells, including their microstructure, in a region of the skin tissue approximately 30 × 30 × 40 µm in size (figure 4). Because of computational size, we were constrained to the near field for the larger three-dimensional calculation.

### 2.4. Camouflage model

**Figure 5.**
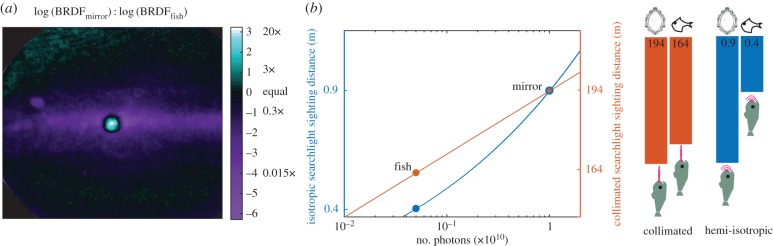
Visibility of hatchetfish skin in the oceanic midwater. (*a*) Ratio of BRDFs for a metallic mirror and the hatchetfish. The pattern in the plane of the page is defined in the same manner as figure 2*a*, while the intensity axis represents the ratio of the BRDF intensity of a metallic mirror versus that of the hatchetfish skin. The ratio of the brightest point in the hatchetfish BRDF compared with the mirror BRDF represents the relative brightness of light returning to a searchbeam predator's eye. (*b*) Relationship of sighting distance as a function of number of photons emitted from a searchlight, shown for both a completely collimated searchbeam (red curve) and a hemi-isotropic searchbeam (blue curve). The circle at the intersection of the two curves represents the sighting distance of a mirror given a searchlight intensity of 10^10^ photons. The red and blue circles show the sighting distance of the hatchetfish given a 20-fold reduction in intensity of the directly reflected beam, as shown in (*a*). The orange and blue bars represent absolute reduction in sighting distance of the hatchetfish relative to a mirror for a searchlight intensity of 10^10^ photons.

To understand the ecological context of this complex scattering structure, we estimated the visibility of hatchetfish skin when illuminated by a predator's bioluminescent searchlight and/or by ambient environmental radiance, as compared with a completely specular surface. To do this, we first estimated the BRDF for a searchlight beam reflected from a flat mirror. We then normalized our experimental hatchetfish BRDF data such that the sum of intensities of all the pixels in the hatchetfish BRDF measurement was equal to that of the mirror measurement. That is, we assumed that the same total amount of light is scattered back to the plane of a camera aperture in both cases, and examined differences in the spatial distribution of this backscattered light and the implications for visibility (figure 5*a*).

With this information, we then derived a modified form of Warrant's bioluminescence threshold detection equation to estimate the fish's sighting distance, *r*, or the distance at which the hatchetfish becomes visible via reflected bioluminescence to a searchlight predator [8]. We compared the hatchetfish's sighting distance with the sighting distance of a metallic mirror (figure 5*b*). In its original formulation, Warrant's equation models the sighting distance of an isotropic bioluminescent point source in ocean water. In our version, we reworked this equation to represent either an isotropic or a collimated source emitted from a predator and reflected off the hatchetfish's back to the predator's eye. The model assumes that the searchlight source is located at a negligibly small angular separation from the predator's eye, and that the hatchetfish's flank has a diameter of 2 cm, close to the diameter of the searchlight beam reaching the fish.

For a hemi-isotropic predatory searchlight (i.e. an isotropic source emitted from a reflective plane), the number of photons, *N*, absorbed by the predator's retina after scattering off the hatchetfish prey is:2.1
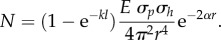


Here, *σ_p_* is an estimated predator pupil diameter cross-sectional area of 4.2 × 10^−5^ m^2^; *σ_h_* is the approximate size of the searchbeam intersecting the hatchetfish's body, estimated to be 3.4 × 10^−4^ m^2^; *r* is the distance between the hatchetfish and the predator's eye; *E* is the number of photons in the bioluminescent flash; *α* is the attenuation coefficient of seawater, estimated to be 0.05 m^−1^; and we assume (1 − e^−*kl*^) = 1 (meaning a predator photoreceptor absorbs all light incident upon it) [8]. We also used a flash detection threshold of five photons absorbed by the retina [37,38]. These values apply to the lower portion of the mesopelagic zone, the hatchetfish's deepest residence. This deepest case presumably finds the minimum searchbeam predator sighting distances. This is because, in brighter, shallower water, the sighting distance of a hatchetfish not already visible in ambient light that is then illuminated by a searchbeam will presumably increase as the contrast of the same reflected flash against the background is decreased.

If, instead, the predatory searchlight is perfectly collimated by a lens and normally incident on the target, the following estimation of sighting distance applies:2.2
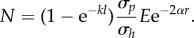


Making the assumptions stated above, equations (2.1) and (2.2) can be simplified to:2.3
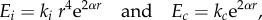
where *E_i_* and *E_c_* are the photons emitted from the predator's isotropic or collimated bioluminescent source, respectively; *α* *=* 0.05 m^−1^; and the remaining variables are combined into the constants *k_i_* = 1.5 × 10^10^ m^−4^ and *k_c_* = 37.5. We used these relationships between the number of photons in a bioluminescent flash and sighting distances *r* to calculate sighting distances (figure 5*b*). To scale between the reflective behaviour of a metallic mirror and the more complex scattering behaviour of the hatchetfish flank, we used the observed decrease in intensity of the scattered peak in the hatchetfish at the specular angle compared with that of a mirror as measured previously by the marine reflectance apparatus [14]. These two cases, of an isotropic searchlight and a completely collimated one, will then place bounds on the biologically realistic case in which a predatory searchlight is perhaps partially focused by a transparent lens-like layer over the surface of the isotropic bioluminescent tissue. However, since evidence suggests that the transparent window tissue of predatory searchlights does not actually serve a strong refractive function, we expect that the realistic sighting distances in the hatchetfish system will be much closer to the hemi-isotropic case than to the fully collimated case [39].

### 2.5. Optical characterization

**Figure 6.**
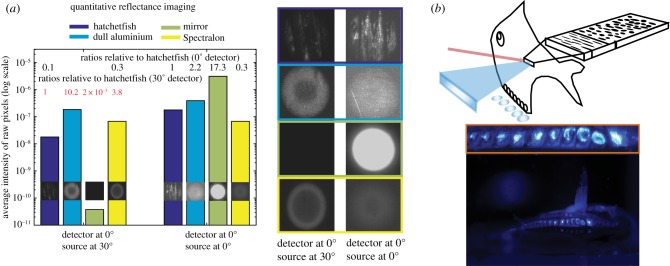
Visibility of various non-absorbing surfaces illuminated by a collimated searchbeam. (*a*) Bar graphs showing average intensities of pixels in exposure-normalized images of a set of non-absorbing surfaces of different BRDF. Left set shows images and average pixel intensities of the surfaces with the source incident normal to the surface and the detector viewing at a 30° angle from normal to the surface. Right set shows the image that is formed when both the detector and the source are pointed normal to the surface. Hatchetfish is shown in dark blue, dull aluminium is shown in light blue, a mirror is shown in green, and Spectralon is in yellow. Central section shows enlarged detail of images in the bar graphs. Surface types are indicated by the colours of the image borders as in left section. (*b*) Top, schematic of overall fish scattering showing how a searchbeam (red arrow) is backscattered lateral to the fish (blue wedge) as well as ventrally through the photophores (blue circles). Bottom, photograph with inset detail (orange box) of a beam incident on the lateral flank of the fish exiting the fish through the ventral photophores.

To experimentally probe the interaction of a collimated bioluminescent searchlight with hatchetfish skin, we illuminated hatchetfish and a set of control surfaces (mirror, unpolished aluminium and Spectralon) with lens-collimated light from a fibre-coupled 455 nm LED (ThorLabs M455F1). We also imaged the light scattered from hatchetfish and other non-absorbing surfaces with well-characterized BRDFs (a mirror, aluminium foil, and a Lambertian surface) under controlled illumination and viewing conditions while the surfaces were immersed in filtered seawater (figure 6*a*,*b*). The mirror was a small, back-silvered cosmetic mirror, the aluminium foil was from a standard kitchen-grade roll of foil, and the Lambertian surface was a diffuse reflectance standard made of the polymer Spectralon (Ocean Optics WS-1 reflectance standard). We ensured that the distances of the source and the detector from the objects remained constant. These images were captured in RAW format with a Nikon D4S camera fitted with a 60 mm macro lens. All surfaces were immersed in filtered seawater and photographs were taken in a darkroom (figure 6*a*). We also imaged the photophores by focusing the camera at the ventral surface of the fish while the collimated LED beam was aimed at the lateral flank of the fish (figure 6*b*). Additionally, fish skin was imaged using darkfield reflected-light microscopy with a Nikon Eclipse LV100ND compound microscope fitted with the same Nikon D4S camera and a trinocular lens adapter.

After ensuring that no pixels in the images were saturated, we cropped them to the size of the subject, averaged the pixel intensity of the blue channel of the raw images, and normalized the images by exposure time. We then averaged the per pixel intensities of the raw images to estimate the contrast a predator might see when viewing the various illuminated surfaces against a dark background. In this experiment, the collimated illuminating beam was always directed normal to the scattering surface, while the camera lens was pointed either normal to the surface or at a 30° angle from normal to the surface.

We also measured the corresponding reflectance spectra from all these surfaces using a USB-driven fibre-optic spectrometer (Ocean Optics USB2000), a reflectance probe with both illuminating and detecting fibres positioned normal to the sample surface, and the same LED light source. These measurements with a spectrometer were used to validate the results of the photometric techniques described above, and to quantify specular reflection from the mirror, since the direct beam from the mirror saturated the camera's detector using the aperture settings in the photography described above.

## 3. Results

By considering TEM images from the sectioning plane perpendicular to the dorsal–ventral axis of the fish and parallel to the anterior–posterior axis of the fish, we discovered that there are two distinct layers of optical structure in hatchetfish skin (figure 1*c*–*e*). The superficial layer, about 10 µm thick, is composed of cells containing stacked guanine platelets with the stack-normal axis oriented perpendicular to the surface of the fish. The dimensions of these superficial stacks when sectioned in the *X*-plane are approximately 10 µm by 1.5 µm with 3–6 guanine platelets of 100 nm thickness per stack. The deeper layer, which is approximately 20–30 µm thick, contains cells that are elliptical when sectioned in the *X*-plane, with a major axis of about 2 µm and a minor axis of about 1 µm, with 10–20 close-packed guanine crystals, also about 100 nm thick. TEM images from a plane perpendicular to the anterior–posterior axis of the fish and parallel to the dorsoventral axis (figure 1*f*) reveal that most of the 10–20 guanine crystals visible in cross-section in both the superficial and deep layers extend parallel to the long axes of these cells for lengths of tens of microns. Therefore, for the purposes of optical modelling, it is reasonable to approximate cells in both the superficial and the deep layers as infinite along the long axes of the cells running parallel to the dorsal–ventral axis of the fish.

To understand the bulk scattering from hatchetfish skin cells as the sum of its hierarchically ordered parts, we first calculated the scattering properties of homogeneous ellipsoids and rectangles the size of individual cells in the hatchetfish skin when sectioned in the *X*-plane (figure 2*c*,*d*) in comparison with the size of individual cells when sectioned in the *Y*-plane (figure 2*e*). These two-dimensional FDTD models of simple shapes qualitatively show that both the inner and outer layer cells contribute to the diffuse component of the BRDF that is parallel to the anterior–posterior axis of the fish (figure 2*a*). This is because, in this two-dimensional model, light incident on the aspects of the ellipses in the *X*-plane in both the superficial and the deep layers of the skin exhibits a broadening of the angular distribution of backscattered light in comparison with light incident on cells oriented in the *Y*-plane. We found that both ellipses and rectangles with smaller major axes, consistent with cells found in the deep layer of skin, have broader backscattering lobes. The magnitude of this effect for elliptical shapes is apparent when considering scattering from rectangles of the same size.

Then, we considered scattering from the same two-dimensional cross-sections but including the subwavelength cellular structure (figure 3). More light backscattered from the more detailed structures, presumably due to the multiple internal boundaries inside each cell causing additional reflection. When we combined the layers into a large three-dimensional model of the hatchetfish skin and calculated the backscattering in both the *X* and *Y* directions, we found a similar pattern of anisotropic scattering to the experimental BRDF data in the near field (figure 4). Although computational limitations prevented us from extending this larger calculation to the far field, this three-dimensional analysis is consistent with the experimental BRDF data as well as the two-dimensional models of scattering from simple single cells in either the *X*- or *Y*-plane orientations, further supporting the structural mechanism causing anisotropic scattering we propose.

To further examine the hatchetfish's complicated scattering properties we compared the hatchetfish BRDF with that of a metallic mirror (figure 5*a*). The maximum radiant intensity of the beam reflected from the mirror is approximately 20 times greater than the maximum radiant intensity scattered from hatchetfish skin.

We then used equation (2.2) to estimate the sighting distance of a mirror versus hatchetfish skin when a predator deploys 10^10^ photons from either an isotropic or a collimated bioluminescent searchlight. In the context of a deep-sea predator's eye, the hatchetfish's redistribution of backscattered radiance compared with a mirror decreases the distance at which a predator will first see the hatchetfish from 0.9 m to 0.4 m in the case of an isotropic searchlight, and from 194 m to 164 m in the case of a perfectly collimated searchlight (figure 5*b*). This is because the light scattered from a searchlight beam striking its skin is spread out along the anterior–posterior arc, reducing the contrast of the resulting image at a distance.

We found that, when viewed at 30°, the image of the hatchetfish skin had less contrast than the image of foil (the foil was 10.2 times brighter than the fish) or a Lambertian surface (3.8 times brighter than the fish), but the fish was much brighter than a mirror. In this illumination and viewing condition, the presence of both the fish and the mirror were barely perceptible in the images relative to the aluminium foil and the Lambertian surface. However, when viewed at an angle normal to the surface, the hatchetfish skin had much less contrast than the mirror (the mirror was 17.3 times brighter than the fish). Hatchetfish skin also had less contrast than the foil in this orientation (the foil was 2.2 times brighter). However, the Lambertian surface was less visible than the fish in this orientation (the fish was three times brighter than the white surface). Interestingly, foil was more visible than the fish in both viewing conditions, although the BRDF of foil is closer to that of the hatchetfish than the other two surfaces [40].

We also imaged the ventral surface of the fish when the lateral flank was illuminated with a collimated beam incident normal to the flank, as described above. Interestingly, light was emitted from the fish's ventral photophores in this orientation (figure 6*b*). The total light reaching the camera in this image from the photophores was about 20% of the light reflecting off the lateral flank of the hatchetfish.

## 4. Discussion

We show a novel, two-component light-scattering structure in hatchetfish skin composed of a superficial layer of broadband dielectric stacks roughly rectangular in cross-section with long axes running parallel to the dorsal–ventral axis of the fish, and a deeper layer of elliptical bundles of high-index material that also have long axes running parallel to the dorsal–ventral axis of the fish (figure 1). When radiance is incident on the lateral flank of the fish, the fish skin exhibits diffuse, broadband backscattering that is markedly concentrated in an arc parallel to the anterior–posterior axis of the animal, independent of the angle at which a ray of light strikes the skin ([14], figure 2). The same composite skin structures also scatter light ventrally, such that, when the lateral side of the animal receives normally incident illumination, some of this light exits the fish body through the ventral counterilluminating photophores. This complex pattern of light scattering is a marked departure from the assumption of specular, mirror-like reflectance leading to camouflage against radially symmetric ambient radiance that is often invoked in midwater ecology [6,41].

Our two-dimensional FDTD calculations provide some insight into the physical origins of this scattering pattern. When we modelled a two-dimensional cross-section of a rectangular cell with a 7 µm width from the superficial layer of the fish skin, the structure exhibited a broad angular scattering distribution similar to that observed in BRDF. When we modelled the two-dimensional cross-sections of the smaller elliptical bundles from the deeper layer, the angular distribution of backscatter was even broader. This pattern was reversed when we considered scattering from very high aspect ratio ellipses and rectangles with a length of 25 µm and width of 1.28 µm. In this cross-section, consistent with scattering from the very long axes of both layers of cells in the *Y*-plane, the angular width of backscatter was much narrower and the amount of light scattered back greater. This pattern is consistent with the outer layer cells being primarily responsible for the specular reflection observed in the BRDF data. Then, for light that penetrates these outer layer cells, the relatively wider backscattering of inner layer cells allows light to diffuse further along the *X*-plane direction before exiting the tissue, consistent with the arc observed in the BRDF data (figure 2). The sum of near-specular reflection from the outer layer and diffusion weighted along the *x*-axis within the inner layer seems to account for the ‘specular arc’ scattering pattern observed as the BRDF from the illuminated flank of the fish.

More detailed calculations that considered the subwavelength structure of the fish skin cells reinforce these notions. Light incident normal to an *X*-plane cross-section of the outer-layer cells is equally forward- and back-scattered. Any light forward-scattered from these outer-layer cells then reaches the elliptical bundles of the inner layer, which are much more isotropic, especially when their subcellular structures are included.

Since the phase functions in figure 3 can only be used to infer scattering properties along the *X*-axis, we also calculated backscattering in the far field from two-dimensional ellipses and rectangles with much larger major axes as a simple approximation of the single cells in the *Y* direction. Ellipses with higher aspect ratios and lengths approaching infinity with respect to the wavelength of light have a narrower backscattering peak. Our models therefore show that the anisotropic shape of the individual cells and their relative orientations in both layers are responsible for the anisotropic scattering. We also modelled the scattering from a small section of hatchetfish skin in three dimensions using FDTD. This model could only be completed in the near field; however, it shows the same qualitative trend as the BRDF data and two-dimensional models.

In the region of the midwater where hatchetfish are found, solar light is dim and primarily downwelling, and predatory bioluminescent searchlights are common. Therefore, for complete camouflage, organisms must deal with both light sources, light from the Sun and bioluminescent light from other animals. The scattering behaviour of hatchetfish scales may provide insight into multimodal camouflage mechanisms in this realm. The ability of hatchetfish skin to scatter the same radially symmetric pattern into the water for any given direction of illumination may be important for hiding against searchlight predators, while also maintaining the advantages of mirror-like reflectance for hiding against radially symmetric angular light. At the same time, predators lurk under their prey, looking for silhouettes against downwelling light [10]. In response, hatchetfish have bioluminescent organs pointing downwards, eliminating the silhouette that forms directly under them [10]. Considering the difference in intensities between the downwelling light and side-welling light, a diffuse reflectance arc along the anteroposterior axis will be less conspicuous than, for example, a diffuse scattering arc along the dorsal–ventral axis of the fish.

When the lateral flank of the fish is illuminated, scattering within the inner layer is strongest along the anterior–posterior axis, but also diffuses downwards within the inner layer in the dorsal–ventral direction. This downward-scattered light is coupled to the ventral-pointing photophores responsible for counter-illumination (figure 6). As a result of this process, about 20% of the light impinging horizontally on the flank of the fish ultimately radiates downwards through the ventral-directed photophores. This result suggests that another function of the complex structures found in the skin may be to redirect ambient or searchbeam light ventrally, re-routing both ambient and predatory light in the direction of the bioluminescent counter-illumination emitted from the photophores.

Given the realistic ranges of possible bioluminescent searchbeam intensities, searchbeam pulse durations and environmental variation in radiance, an individual fish could plausibly encounter scenarios in different portions of its habitat in which ventrally re-directed light could be dimmer than, about the same intensity as, or much brighter than downwelling radiance. However, sending additional horizontal light downwards (downwelling is always the radiance of greatest intensity in the midwater) will always be the least visible and therefore least harmful option available to the fish, no matter the environmental radiance or predatory context. Therefore, we predict that, at one extreme (relatively shallow water with comparatively isotropic ambient radiance), this downward re-direction of ambient light may actively aid camouflage by reducing the intensity otherwise required from the photophores. At the other extreme (deep water with extremely anisotropic radiance, low luminance and searchbeams of high intensity), the structures may function as a ‘beam dump’, i.e. a least harmful option for handling bright predatory searchbeams that redirects them away from the emitting predator and downwards in the direction of least resulting image contrast.

Recent work in the squid *Galiteuthis* suggests that light guiding can result from cellular arrays of quasi-ordered parallel dielectric structures similar to those we report here in the hatchetfish [30]. We speculate that an effect similar to that in the *Galiteuthis* photophore acts within the inner layer to direct stray light downwards through the flank to the photophores. We speculate that an emerging theme in midwater camouflage is of using quasi-ordered subwavelength-scale vertical structures to effect a strategy of ‘when in doubt, send light down’, since, as discussed above, in all midwater ecological contexts this will be the least damaging option.

Together, our data suggest that the composite refractive structure in hatchetfish skin increases camouflage from all viewing angles when illuminated either by ambient light and/or by a predator's searchlight. The reflectance from the scales increases the sighting distance to a searchlight predator (viewing at 0° relative to the beam) as well as from detection in ambient light (viewing at 30° relative to the beam) when compared with other potential scattering mechanisms the fish might have evolved. At the same time, any light incident on the flank is also redirected downwards through the line of ventral photophores, providing hiding power against downwelling illumination to any upward-looking predators. In contrast, a specular fish would produce a bright spot visible from several viewing angles when illuminated with a searchlight, and a Lambertian fish would be visible in ambient light, while neither of these other possible reflective hiding scattering strategies would result in light being directed downwards through the ventral photophores. Although we have not included a quantitative assessment of the fish's ability to be camouflaged in the horizontally radially symmetric ambient light of the midwater, qualitatively, the fact that the fish's BRDF is also close to horizontally radially symmetric suggests that it would remain hidden when exposed to radially symmetric ambient light.

Here we have described a novel photonic structure in a deep-sea fish, and provided initial insight into its evolved role in deep pelagic camouflage where dim downwelling solar radiance and bioluminescent searchlights seem to play equally important roles in visual ecology. The skin's structure also indicates a new method for producing broadband reflectance with an unusual diffuse reflectance pattern.

## Data accessibility

All FDTD codes and images of hatchetfish skin used to quantify camouflage have been uploaded to the Dryad repository, and are accessible at the following link: http://dx.doi.org/10.5061/dryad.31v01 [42].

## References

[1] Denton EJ 1970 Review lecture: on the organization of reflecting surfaces in some marine animals. Phil. Trans. R. Soc. Lond. B 258, 285–313. (10.1098/rstb.1970.0037) 22408830

[2] Jordan TM, Partridge JC, Roberts NW 2012 Non-polarizing broadband multilayer reflectors in fish. Nat. Photonics 6, 759–763. (10.1038/nphoton.2012.260) 23160173 PMC3496938

[3] Brady PC, Travis KA, Maginnis T, Cummings ME 2013 Polaro-cryptic mirror of the lookdown as a biological model for open ocean camouflage. Proc. Natl Acad. Sci. USA 110, 9764–9769. (10.1073/pnas.1222125110) 23716701 PMC3683730

[4] Johnsen S 2002 Cryptic and conspicuous coloration in the pelagic environment. Proc. R. Soc. Lond. B 269, 243–256. (10.1098/rspb.2001.1855) PMC1690886 11839193

[5] Johnsen S 2003 Lifting the cloak of invisibility: the effects of changing optical conditions on pelagic crypsis. Integr. Comp. Biol. 43, 580–590. (10.1093/icb/43.4.580) 21680466

[6] Johnsen S, Sosik HM 2003 Cryptic coloration and mirrored sides as camouflage strategies in near-surface pelagic habitats: implications for foraging and predator avoidance. Limnol. Oceanogr. 48, 1277–1288. (10.4319/lo.2003.48.3.1277)

[7] Quéro JC, Hureau JC, Post A, Saldanha L, Karrer C 1990 Check-list of the fishes of the eastern tropical Atlantic. Paris, France: UNESCO.

[8] Warrant EJ, Locket NA 2004 Vision in the deep sea. Biol. Rev. 79, 671–712. (10.1017/S1464793103006420) 15366767

[9] You Y, Zhai PW, Kattawar GW, Yang P 2009 Polarized radiance fields under a dynamic ocean surface: a three-dimensional radiative transfer solution. Appl. Opt. 48, 3019–3029. (10.1364/AO.48.003019) 19488114

[10] Johnsen S, Widder EA, Mobley CD 2004 Propagation and perception of bioluminescence: factors affecting counterillumination as a cryptic strategy. Biol. Bull. 207, 1–16. (10.2307/1543624) 15315939

[11] Zylinski S, Johnsen S 2011 Mesopelagic cephalopods switch between transparency and pigmentation to optimize camouflage in the deep. Curr. Biol. 21, 1937–1941. (10.1016/j.cub.2011.10.014) 22079113

[12] Land MF, Osorio DC 2011 Marine optics: dark disguise. Curr. Biol. 21, R918–R920. (10.1016/j.cub.2011.10.009) 22115458

[13] Vukusic P, Stavenga DG 2009 Physical methods for investigating structural colours in biological systems. J. R. Soc. Interface 6(Suppl. 2), S133–S148. (10.1098/rsif.2008.0386.focus) 19158009 PMC2706471

[14] Haag JM, Jaffe JS, Sweeney AM 2013 Measurement system for marine animal reflectance functions. Opt. Express 21, 3603–3616. (10.1364/OE.21.003603) 23481817

[15] Greenstein LM 1973 Pearlescence: the optical behavior of nacreous and interference pigments. Pigment Handb. 3, 357–390.

[16] Levy-Lior A, Pokroy B, Levavi-Sivan B, Leiserowitz L, Weiner S, Addadi L 2008 Biogenic guanine crystals from the skin of fish may be designed to enhance light reflectance. Cryst. Growth Des. 8, 507–511. (10.1021/cg0704753)

[17] Mueller KP, Labhart T 2010 Polarizing optics in a spider eye. J. Comp. Physiol. A 196, 335–348. (10.1007/s00359-010-0516-6) 20229246

[18] Stavenga DG, Leertouwer HL, Wilts BD 2014 The colouration toolkit of the Pipevine Swallowtail butterfly, Battus philenor: thin films, papiliochromes, and melanin. J. Comp. Physiol. A 200, 547–561. (10.1007/s00359-014-0901-7) 24715265

[19] Seago AE, Brady P, Vigneron JP, Schultz TD 2009 Gold bugs and beyond: a review of iridescence and structural colour mechanisms in beetles (Coleoptera). J. R. Soc. Interface 6(Suppl. 2), S165–S184. (10.1098/rsif.2008.0354.focus) 18957361 PMC2586663

[20] Jordan TM, Partridge JC, Roberts NW 2014 Disordered animal multilayer reflectors and the localization of light. J. R. Soc. Interface 11, 20140948 (10.1098/rsif.2014.0948) 25339688 PMC4223918

[21] Burresi M, Cortese L, Pattelli L, Kolle M, Vukusic P, Wiersma DS, Steiner U, Vignolini S 2014 Bright-white beetle scales optimise multiple scattering of light. Sci. Rep. 4, 6077 (10.1038/srep06075) 25123449 PMC4133710

[22] Holt AL, Sweeney AM, Johnsen S, Morse DE 2011 A highly distributed Bragg stack with unique geometry provides effective camouflage for Loliginid squid eyes. J. R. Soc. Interface 8, 1386–1399. (10.1098/rsif.2010.0702) 21325315 PMC3163417

[23] Ghoshal A, DeMartini DG, Eck E, Morse DE 2013 Optical parameters of the tunable Bragg reflectors in squid. J. R. Soc. Interface 10, 20130386 (10.1098/rsif.2013.0386) 23740489 PMC4043173

[24] McKenzie DR, Yin Y, McFall WD 1995 Silvery fish skin as an example of a chaotic reflector. Proc. R. Soc. Lond. A 451, 579–584. (10.1098/rspa.1995.0144)

[25] Bell GR, Mäthger LM, Gao M, Senft SL, Kuzirian AM, Kattawar GW, Hanlon RT 2014 Diffuse white structural coloration from multilayer reflectors in a squid. Adv. Mater. 26, 4352–4356. (10.1002/adma.201400383) 24789321

[26] Warrant E 2000 The eyes of deep-sea fishes and the changing nature of visual scenes with depth. Phil. Trans. R. Soc. Lond. B 355, 1155–1159. (10.1098/rstb.2000.0658) 11079389 PMC1692855

[27] Warrant E 2004 Vision in the dimmest habitats on Earth. J. Comp. Physiol. A 190, 765–789. (10.1007/s00359-004-0546-z) 15375626

[28] Johnsen S 2005 The red and the black: bioluminescence and the color of animals in the deep sea. Integr. Comp. Biol. 45, 234–246. (10.1093/icb/45.2.234) 21676767

[29] Childress JJ, Barnes AT, Quetin LB, Robison BH 1978 Thermally protecting cod ends for the recovery of living deep-sea animals. Deep Sea Res. 25, 419IN5421–420IN6422. (10.1016/0146-6291(78)90568-4)

[30] Holt AL, Sweeney AM 2016 Open water camouflage via ‘leaky’ light guides in the midwater squid Galiteuthis. J. R. Soc. Interface 13, 20160230 (10.1098/rsif.2016.0230) 27278362 PMC4938086

[31] Taflove A, Hagness SC 1995 Computational electrodynamics: the finite-difference time-domain method, 2nd edn Norwood, MA: Artech House.

[32] Zhao S, Brady PC, Gao M, Etheredge RI, Kattawar GW, Cummings ME 2015 Broadband and polarization reflectors in the lookdown, Selene vomer. J. R. Soc. Interface 12, 20141390 (10.1098/rsif.2014.1390) 25673301 PMC4345507

[33] Oskooi AF, Roundy D, Ibanescu M, Bermel P, Joannopoulos JD, Johnson SG 2010 MEEP: A flexible free-software package for electromagnetic simulations by the FDTD method. Comput. Phys. Commun. 181, 687–702. (10.1016/j.cpc.2009.11.008)

[34] Herring PJ 1994 Reflective systems in aquatic animals. Comp. Biochem. Physiol. A Physiol. 109, 513–546. (10.1016/0300-9629(94)90192-9)

[35] Levy-Lior A, Shimoni E, Schwartz O, Gavish-Regev E, Oron D, Oxford G, Weiner S, Addadi L 2010 Guanine-based biogenic photonic-crystal arrays in fish and spiders. Adv. Funct. Mater. 20, 320–329. (10.1002/adfm.200901437)

[36] Gur D, Politi Y, Sivan B, Fratzl P, Weiner S, Addadi L 2013 Guanine-based photonic crystals in fish scales form from an amorphous precursor. Angew. Chem. Int. Ed. Engl. 125, 406–409. (10.1002/ange.201205336) 22951999

[37] Denton EJ 1990 Light and vision at depths greater than 200 metres. In Light and Life in the Sea (eds PJ Herring, AK Campbell, M Whitfield, L Maddock), pp. 127–148. Cambridge, UK: Cambridge University Press.

[38] Warrant EJ 1999 Seeing better at night: life style, eye design and the optimum strategy of spatial and temporal summation. Vision Res. 39, 1611–1630. 10343855 10.1016/s0042-6989(98)00262-4

[39] Herring PJ, Cope C 2005 Red bioluminescence in fishes: on the suborbital photophores of Malacosteus, Pachystomias and Aristostomias. Mar. Biol. 148, 383–394. (10.1007/s00227-005-0085-3)

[40] Dana KJ, Van Ginneken B, Nayar SK, Koenderink JJ 1999 Reflectance and texture of real-world surfaces. ACM Trans. Graph. 18, 1–34. (10.1145/300776.300778)

[41] Johnsen S, Gassmann E, Reynolds RA, Stramski D, Mobley C 2014 The asymmetry of the underwater horizontal light field and its implications for mirror-based camouflage in silvery pelagic fish. Limnol. Oceanogr. 59, 1839–1852. (10.4319/lo.2014.59.6.1839)

[42] Rosenthal EI, Holt AL, Sweeney AM 2017 Data from: three-dimensional midwater camouflage from a novel two-component photonic structure in hatchetfish skin. *Dryad Digital Repository*. (10.5061/dryad.31v01) PMC5454286 28468923

